# Supplementary material for: Insights into Reactive Oxygen Species Production-Scavenging System Involved in Sugarcane Response to Xanthomonas albilineans Infection under Drought Stress
Source: Plants (Basel). 2024 Mar 17;13(6):862. doi: 10.3390/plants13060862 (PMC10974620; doi:10.3390/plants13060862)
Supplement: Supplementary file 1 [file plants-13-00862-s001.zip › plants-2884704-supplementary.pdf]

Table S1. Primer pairs used to analyze *ScRBOHD*, *ScSOD*, and *ScCAT* gene expressions by qRT-PCR assay.

**Target Gene**	**Primer name**	**Sequence (5'→3')**	**Amplification condition**
ScRBOHD	q-ScRBOHD-F	GACAAAGGAGCTGCGTGAAC	95 °C, 30 s; 95 °C, 10 s, 60 °C, 30 s, 40 cycles; 95 °C, 15 s, 60 °C, 60 s, 95 °C, 15 s
	q-ScRBOHD-R	CCTGCCGTCTACATCCATCC	
ScSOD	q-ScSOD-F	TCCCACTAACTGGGCCAAAC	
	q-ScSOD-R	TCGTGACCACCCTTTCCAAG	
ScCAT	q-ScCAT--F	TCCAGCTTCTGGACCACCAA	
	q-ScCAT--R	CGTTCCCTGTCGAACTGAGC	
ScGAPDH	GAPDH-F	CACGGCCACTGGAAGCA	
	GAPDH-R	TCCTCAGGGTTCCTGATGCC	
